# Preface: paving the road for AI in molecular sciences

**DOI:** 10.1093/nsr/nwae010

**Published:** 2023-12-29

**Authors:** Yi Qin Gao

**Affiliations:** Beijing National Laboratory for Molecular Sciences, College of Chemistry and Molecular Engineering, Peking University, China

During the past decade, the burgeoning development of artificial intelligence (AI), particularly deep learning, has resulted in an impressive and even superhuman performance on many challenging tasks such as computer vision and natural language processing. Deep learning is also transforming many areas in science, and in particular, has great potential in molecular sciences. It has already shown great power in tasks such as protein structure prediction, and protein and material design. In the long run, AI has the potential to reshape or even re-define chemical research. However, unlike the mature deployment of deep learning in computer vision and natural language processing, its development in chemistry is still at an early stage, largely because the inductive biases of molecules are completely different from those of images or texts. The lack of standardized data and underdeveloped model also play a role in the relatively limited usage of deep learning in chemical sciences. However, such a situation is changing with a remarkable acceleration and inclusion of AI tools and deserves serious consideration for researchers working in many different areas of chemistry.

This Special Topic aims at highlighting some of the latest research advances in the applications of AI in the chemistry studies. The review article by Bina Fu and Dong Hui Zhang [1] summarizes the development of the fundamental invariant-neural network (FI-NN) approach in representing highly accurate potential energy surfaces, which has the potential to achieve accurate quantum dynamics studies of complex molecular systems. Wei Li, Guoqiang Wang, and Jing Ma [2] reviewed the recent advances made to discover patterns hidden behind extensive computational and experimental data with physical-informed and brain-inspired ML, to aid in the exploration of structure-property relationships, and accelerate calculations of energy, force, and other properties. In the Perspective article by Shuo Feng, Aoran Cai and Yang Wang *et al.* [3], they proposed a robotic AI-Chemist which is characterized by high-throughput data acquisition, interactive calibration of theoretical and experimental data, and validation of literatures, with the aim to establish an AI-ready database covering massive scientific data and integrating chemical knowledge. The Review article by Wen Jun Xie and Arieh Warshel [4] focuses on how to employ generative AI for enzyme sequence analysis as well as enzyme engineering, which is expected to significantly enhance our knowledge of enzymes and expedite the creation of superior biocatalysts.

As the guest editor, I would like to express our sincere appreciation to all the authors, reviewers and also the editorial office of *NSR* for their efforts to make this special topic possible. I hope that this special topic will gain broad attention from chemistry, physics, materials science and other related fields.

***Conflict of interest statement***. None declared.
